# Supplementary material for: Understanding effectiveness of a low-cost food package for ensuring food security during the COVID-19 at the household level: Difference-in-differences analyses of a quasi-experimental trial in Bangladesh
Source: PLoS One. 2026 Mar 13;21(3):e0344609. doi: 10.1371/journal.pone.0344609 (PMC12987448; doi:10.1371/journal.pone.0344609)
Supplement: Attachment — Submitted filename: Response to Reviewers.pdf [file pone.0344609.s003.pdf]

Rebuttal letter (PONE-D-22-31934R1)

Dear editorial team members, dear reviewers,

We very much appreciate the thorough, thoughtful, and constructive reviews we received for the manuscript "*Understanding the effectiveness of a low-cost food package for ensuring food security at the household level during the COVID-19 pandemic: Difference-in-differences analyses of a community-based quasi-experimental trial in Bangladesh*". We also greatly appreciate the invitation to resubmit the revised manuscript. Below, we describe in detail how we have addressed each of the points raised by the editors.

Thank you very much.
Corresponding author.

Additional Editor Comments:

1. It is a valuable study that can be made more useful by considering a few points.
Title: Since the project's findings are limited to a country, it is better to mention Bangladesh in the title.
Response. Thank you so much for the valuable suggestion. We have added it accordingly.

2. Abstract: How was the food security status was measured?
Response. We used Household Food Insecurity Access Scale and mentioned it in the Abstract.

3. Introduction: Previous studies on the provision of food aids by international organizations in Bangladesh such as WFP are not mentioned.
Response. Thanks for your valuable suggestions. we have added the information in the introduction section.

4. Methods: The amount of macro- and micronutrients provided by the package is not presented.
Response. Following the dietary guidelines for Bangladesh, we didn’t provide any separate micro and macronutrients in the package. However, Nutritional value of the food package were calculated using the food composition table. Table 1 details the energy content of the food package.

5. Eligibility criteria for low-cost food package is not clear.
Response. Thanks for highlighting the issue. We have discussed the food package selection process in the intervention section. We have considered the World Health Organization recommended energy requirement for an adult person is 2420 Kcal per day, and for a child is 1290 Kcal for finalising the food amount and technical expert group report of Bangladesh government to finalise the food items.

6. There is no reference in the following sentence: “We used the Household Food Insecurity Access Scale to calculate the Household Food Insecurity Access Prevalence (HFIAP) based on their responses on the level of access to food and according to their score (Ref).”

Response. We have added the reference accordingly. Please see reference no 32. Thanks for pointing this out.

7. Measuring socio-economic variables were not mentioned.
Response. Thanks for the suggestion. We have added accordingly.

8. Results: The figures in the tables are repeated in the text.
Response. Thanks for your comment on it. Following you comment we have re-write the section accordingly.

9. Discussion: The main outcome variable of food security is different in two intervention and control groups. Researchers need to discuss whether it influenced the study results and whether there was a need for matching at the beginning of the study.
Response. Thanks for mentioning the issue. We have discussed the issue accordingly in the limitation section

10. No findings were presented about the following discussion:m"We found similar coping strategies in urban and rural areas in the baseline data. Some households received different types of support from various sources,…"

Response. Thanks for the comment. We have added the findings in the result section.
