# Supplementary material for: Understanding effectiveness of a low-cost food package for ensuring food security during the COVID-19 at the household level: Difference-in-differences analyses of a quasi-experimental trial in Bangladesh
Source: PLoS One. 2026 Mar 13;21(3):e0344609. doi: 10.1371/journal.pone.0344609 (PMC12987448; doi:10.1371/journal.pone.0344609)
Supplement: Attachment — Submitted filename: Rebuttal letter .docx [file pone.0344609.s002.docx]

Rebuttal letter (PONE-D-22-31934)

Dear editorial team members, dear reviewers,

We very much appreciate the thorough, thoughtful, and constructive reviews we received for the manuscript "*Understanding the effectiveness of a low-cost food package for ensuring food security at the household level during the COVID-19 pandemic: Difference-in-differences analyses of a community-based quasi-experimental trial*". We also greatly appreciate the invitation to resubmit the revised manuscript. Below, we describe in detail how we have addressed each of the points raised by each of the Referees.

Thank you very much.

Corresponding author.

**Reviewer #1:** Thanks very much for the opportunity to review this interesting article. The development of a low-cost food basket is a much-needed intervention in the face of COVID-19 and other global threats, and evaluation of such an effort is critical. While factors contributing to the creation of the intervention is shared, more detail as to the targeting and overall approach would be valuable.

**Response:** Thank you so much for your comment. We have updated the methodology section accordingly.

The methodological details are very strong in some areas but omits key information. For example, there is not a clear picture of how control and intervention groups were sorted. This is particularly glaring because they are unbalanced in the outcome variable of interest at baseline. It’s possible that differences in findings thus are attributable to their initial differences in starting points, as opposed to the intervention. This point is not discussed and should be identified in the text as a key shortcoming of this study.

**Response:** Thank you so much for your suggestions. We have added details information about the participants selection in the methodology section (selection of the study participants). Also mentioned the possible shortcomings in the limitations section.

Additional comments are included as follows:

Check presentation of the findings in the abstract carefully, will be important to address methods used more clearly.

**Response:** Thank you for the comment. We have made the changes accordingly in the methodology section of abstract and main text as well.

Check for spelling errors in the abstract (eg, improv for impove).

**Response:** Thank you for the suggestion. We have Checked and corrected the spelling.

The writing throughout is organized into very long paragraphs. It would be more digestible if the paragraphs were more targeted.

**Response:** Noted. We have re-articulated the paragraph throughout the manuscript.

Introduction: There has been a lot of food insecurity data about the pandemic available at this point; studies in Britain are not the most relevant to the Bangladesh context. More global and Bangladeshi (if available) studies would be better context.

**Response:** Thank you so much for your valuable suggestion. We have incorporated information from some other studies from the similar context.

Orienting the introduction on acute food insecurity in disasters and the ways such a food package might have broader relevance to other scenarios (even beyond epidemic/pandemic) would be a valuable approach.

**Response:** Thank you for your suggestion. We have highlighted this issue in introduction section.

4 adults and 1 child is an odd target for the intervention given that’s not likely the average structure of households. How and why was this selected?

**Response:** Thank you for the queries. Prior to selecting the HH for intervention and control group, we conducted a rapid census in the selected geographic location. This data indicated that the median of adult members (more than 5 years old being counted as adult members) is 4, and the median of children is 1. This data also aligned with the nationally representative statistics in Bangladesh. The Bangladesh National Household Income and Expenditure survey indicated that the size of the average HH in Bangladesh is 4.2.

This was paired with extensive outcome measures for children, but if the intervention is only targeted to one child in the household how was this addressed?

**Response:** Thank you so much for bring this issue into light. Our household census data indicated that only a few (2 out of 157) households had more than one child at the time of the census. For this reason, we have designed the food package for one child and didn’t modify the food package for the additional children.

The study design is not adequately described. How were households determined to be intervention or control households? Was this randomized?

**Response:** We have added a section titled “selection of the study participants” where participant selection process is described in detail. This section indicated that participants had missed any meal during last month due to not having food in the household, have lost their livelihood due to the pandemic, had a monthly income less than or equal to 15000 BDT and were willing to provide their consent to participate were included into the study.

Selection criteria of food insecurity is included, but then households that are food secure are in the sample.

**Response:** Thank you for bringing up the issue. During household listing, we asked some specific questions to determine if households had missed any meals in the last month due to lack of food, lost their livelihood due to the pandemic, or had a monthly income that met the pre-defined criteria mentioned in the participant selection section of the manuscript. If they met these requirements, they were included in the sample frame for both arms of the study. It's important to note that the food security status of participating households was assessed with the HFIAS tool after they were enrolled in the study.

Substantial discussion of the uneven sample is needed – far more individuals were in the food insecure category for the intervention group, allowing for a great potential impact if the intervention is most effective for the severely food insecure. The limitations fail to discuss this.

Response: Thank you so much for bring the issue in light. We have mentioned this issue in the limitations section.

Reviewer #2: Editing is necessary. Abstract: More information on selecting intervention and control group must be presented.

**Response:** Thank you so much for the valuable suggestion. We have added the information in the abstract section.

Introduction: There are no literature on the effectiveness of food baskets to improve food insecurity in Bangladesh or other countries.

**Response:** Thank you for raising the issue. We already had some information in the discussion section. However, according to your suggestion, we have added some more information in the introduction and discussion section.

Methods: Considerations for developing food package needs references.

**Response:** Thank you for the query. We have discussed the whole procedure under the intervention section and added reference (ref 24).

Which criteria was used to select the control group?

**Response:** We have added the participant selection section in the main text and selection of control group has been discussed in detail in this section.

How many months did the intervention last?

**Response:** We provided the intervention for three months Thank you so much for the comment. We added this information is in the intervention section of the main text.

We used the Household Food Insecurity Access Scale to calculate the Household Food Insecurity Access Prevalence (HFIAP) based on their responses on the level of access to food and according to their score (Ref???).

**Response:** Thank you for addressing the issue. We have updated the reference.

Repeated measure ANOVA is recommended to detect the differences between intervention and control group adjusted for other variables.

**Response:** Thank you so much for the comment. We applied difference-in-differences analysis to measure the effect of the intervention as the differences between intervention and control group that was adjusted for other variables.

Results: The text of the findings is a repetition of the numbers in Tables and figures. Only significant differences are important to mention.

**Response:** Thank you so much. We have reviewed the results section and edited accordingly.

Figures 1 and 2 were not accessible.

**Response:** Thank you so much. We have updated the figures.

Discussion: Some discussions are unrelated to the results: “We found similar coping strategies in urban and rural areas in the baseline data. Some households received different types of support from various sources, but no systematic relief or safety net program was implemented in those areas.”

**Response:** Thank you so much. We have updated the text.

Comparisons of effectiveness with other studies have not been done.

**Response:** Thank you for your feedback. Unfortunately, we didn’t find any relevant study to compare during the manuscript preparation. That’s why we couldn’t compare the effectiveness of our intervention with other studies.

Acknowledgement: The authors would like to thank all the participants and their parents…???

**Response:** Thank you so much for addressing the issue. We have corrected the typo.
