# Supplementary material for: Understanding effectiveness of a low-cost food package for ensuring food security during the COVID-19 at the household level: Difference-in-differences analyses of a quasi-experimental trial in Bangladesh
Source: PLoS One. 2026 Mar 13;21(3):e0344609. doi: 10.1371/journal.pone.0344609 (PMC12987448; doi:10.1371/journal.pone.0344609)
Supplement: Attachment — Submitted filename: Response to reviewer V2.docx [file pone.0344609.s004.docx]

1. **Title**: Since the study is based solely in Bangladesh, the title should reflect this geographical scope.

Response: Thank you so much for the valuable suggestion. We have added it accordingly. Please see line 3 in the manuscript file.

2. **Abstract**: Please specify how food security status was measured.

Response: We used Household Food Insecurity Access Scale and mentioned it in the Abstract. Please see line 27-28 in the manuscript file.

3. **Introduction**: Include a brief overview of previous food aid interventions in Bangladesh, particularly by international organizations such as the WFP, to contextualize your study.

Response: - thanks for your valuable suggestions. we have added the information in the introduction section. Please see line: 74 to 76 where the information is included.

4. **Methods**:
   - Provide details on the **macro- and micronutrient content** of the food packages.

Response: following the guideline we didn’t provide any separate micro and macronutrients in the package. However, Nutritional value of the food package was calculated using the food composition table. Please see table 1 for details

- Clarify the **eligibility criteria** used to determine who received the food packages.: Thank you for the comment. We discussed the eligibility criteria in the selection of the study participant’s section. Please see line 91-95 in the manuscript.
- Provide a reference for the use of the **Household Food Insecurity Access Scale (HFIAS)**, which is currently missing.: Thanks for sharing the concern. We have added accordingly. Please see reference no 32.
- Include a description of how **socio-economic variables** were measured and analyzed.: Thanks for the suggestion. We have added accordingly in the data analysis section

5. **Results**: Avoid repeating detailed figures in both the tables and the narrative text.: Thanks for your comment on it. Following you comment we have re-write the section accordingly.
6. **Discussion**:
   - Address the potential impact of baseline differences in food security between intervention and control groups, and whether matching or further statistical control was needed.; Thanks for mentioning the issue. We have discussed the issue accordingly in the limitation section. Please see line 284-287 for the updated writeup.
   - Clarify the source of the statement about coping strategies and support received by households, and ensure relevant findings are presented to support the discussion.: - Thanks for the comment. Coping strategies in the result section are discussed in between 208-210. Also, relevant findings are presented in between lines 241-244 in the discussion section.

Title page

• Title should contain the timing of COVID-19

Response: Added in the title
• Line 5: Mention (icddr,b) after Bangladesh before Dhaka

Response: Made the change
• Line 13: Same as line 5

Response: Made the change
• Line 14: 1206 will be 1212
Response: Made the change

Abstract

• Revise the whole abstract keeping within 300 words and using standard English

Response: we have revised the whole abstract, Shorten it within 300 words and made the changes in writing pattern.

Introduction

• In the first paragraph, the word “worldwide” used several times. Kindly use synonyms.

Response: Made the change in the first paragraph
• Line 61-62: Fonts are different. Kindly make it same throughout the manuscript.

All are changed into Times new Roman (11 pt)

Methods

• Line 86: km2 will be km2

Corrected
• Line 97: Add (,) after participate

Added
• Line 99: households will be HH

Corrected
• Line 145: Replace field research assistants by FRAs

Corrected
• Line 149: Delete “Food Frequency Questionnaire”

Corrected
• Line 158: Report will be reported

• Line 160-161: Replace Household Food Insecurity Access Scale by HFIAS

Corrected
• Line 170: under five will be under-five

Corrected

Results

• Line 210: illustrates will be illustrated

Corrected
• Line 215: indicate will be indicated

Corrected
• Line 221: indicate will be indicated

Corrected

Discussion

• Line 234: show will be showed

Corrected
• Line 238: shot term will be short-term

Corrected
• Line 240: show will be showed

Corrected
• Line 268: Behavioral change communication will be Behavioral Change Communication
Changed accordingly
Acknowledgement

• Check the list of core donors

Updated accordingly
